# Challenges and opportunities towards the road of universal health coverage (UHC) in Nepal: a systematic review

**DOI:** 10.1186/s13690-019-0331-7

**Published:** 2019-02-04

**Authors:** Chhabi Lal Ranabhat, Chun-Bae Kim, Ajanta Singh, Devaraj Acharya, Krishna Pathak, Basundhara Sharma, Shiva Raj Mishra

**Affiliations:** 1 Manmohan Memorial Institute of Health Science, Solteemod, Kathmandu -17, POB 44300 Nepal; 2 Institute for Poverty Alleviation and International Development, Yonsei University, Ilsanro, 162 Wonju Si, Gangwon do Republic of Korea; 3 Institute for Poverty Alleviation and International Development (IPAID) at Yonsei University 1, Yonseidae-gil, Wonju Si, Gangwon-do South Korea; 4 Institute of Medicine, Maharajgunj Nursing Campus, Kathmandu, Nepal; 5 Central Campus, Tribhuwan University, Kritipur, Kathmandu, Nepal; 6 National Tuberculosis Center, Thimi, Bhaktapur, Nepal; 7 Nepal Development Society, Chitwan, Nepal

**Keywords:** Universal health coverage, Opportunity and challenges, Sustainable development goal, Out-of-pocket expenditure, Nepal, Health insurance

## Abstract

**Background:**

Universal health coverage (UHC) assures all types of health service and protects all citizens financially in any conditions due to illness. Globally, the UN sustainable development goal (SDG) provides high priority for UHC as a health related goal. The National health system of Nepal has prioritized in similar way. The aim of this study is to explore the challenges and opportunities on the road to UHC in Nepal.

**Method:**

We used varieties of search terminologies with popular search engines like PubMed, Google, Google Scholar, etc. to identify studies regarding Nepal’s progress towards UHC. Reports of original studies, policies, guidelines and government manuals were taken from the web pages of Ministry of Health and its department/division. Searches were designed to identify the status of service coverage on UHC, financial protection on health particularly, health insurance coverage with its legal status. Other associated factors related to UHC were also explored and presented in Preferred Reporting Items for Systematic Reviews and Meta-Analyses (PRISMA) flow chart.

**Results:**

We found 14 studies that were related to legal assurance, risk pulling and financing of health service, 11 studies associated to UHC service coverage status and, 7 articles linked to government stewardship, health system and governance on health care. Constitutional provision, global support, progress on the health insurance act, decentralization of health service to the grass root level, positive trends of increasing service coverage are seen as opportunities. However, existing volunteer types of health insurance, misleading role of trade unions and high proportion of population outside the country are main challenges. The political commitment under the changing political context, a sense of national priority and international support were identified as the facilitating factors towards UHC.

**Conclusion:**

To achieve UHC, service and population coverage of health services has to be expanded along with financial protection for marginalized communities. Government stewardship, support of stakeholders and fair contribution and distribution of resources by appropriate health financing modality can speed up the path of UHC in Nepal.

## Background

Universal health coverage (UHC) is a broad concept that has been implemented in several ways. The common denominator for all such programs is some form of government action aimed at extending access to health care as widely as possible and setting minimum standards. Universal health care should be implemented through legislation, regulation and taxation. In UHC, all people can use the health promotion, prevention, assistance, rehabilitation and palliative care services that they need, in sufficient quality to be effective, while also ensuring that the use of these services does not expose the user to financial hardship [1]. UHC involves three coverage dimensions – health services, finance, and population – and is a dynamic, continuous process that changes in response to shifting demographics, epidemiological and technological trends, as well as people’s expectations [2]. There is an equal importance in quality health service, financial management and assurance of health service with equity and access [3].

Operationally, UHC is defined as legislation provision for universal health insurance and > 90% coverage for skill birth attendance and prepayment health insurance that assures the service coverage with legal guarantees [4]. In this standard, only 58 countries (30.41%) have achieved UHC and almost all are from Organization for Economic Cooperation and Development (OECD) countries, plus some developed and a few developing countries. Germany was the first country to start UHC as a sickness fund and after 2010, a few more countries started as well [5]. Tracking the status of UHC by the World Bank and World Health Organization (WHO) monitoring report 2017, which mentions the service coverage and financial protection situation by country [6]. To measure the status for UHC, there are 16 indicators related to service coverage and two indicators are related to financial protection. A UHC index has been prepared compiling four reproductive, maternal, newborn and child health indicators, four infectious disease control, four non-communicable diseases and four service capacity and access indicators [7]. For financial protection, out of pocket spending and catastrophic health expenditure assessment are indicators [8]. To overcome the financial burden of the entire population during illness without discrimination and quality of health service, the UN emphasized the need to achieve UHC in the sustainable development goal (SDG) in health [9]. UHC is not only contributing to health service, population and financial protection coverage but also significantly increasing life expectancy [10] and reducing adult mortality [11, 12]. This challenging goal to achieve health coverage globally, nationally and sub-nationally is not easy due to many obstacles in health care systems, policy and the political-economic environment.

There is gross inequality in health status between developing and developed countries, poor and rich, male and female and other groups. Beyond health inequalities, approximately 44 million households, or say more than 150 million individuals worldwide, face catastrophic health-care expenditures; of these, about 25 million households containing more than 100 million people are pushed into poverty by these costs [13]. Beyond the different constraints, Nepal has achieved satisfactory public health service coverage (> 85% child vaccine coverage, > 50% skilled birth attendance and significant reduction in communicable diseases) [14]. There are yet many challenges facing the delivery of high-quality medical services without a financial burden to the entire population. More than two-third of the population depend on out-of-pocket expenditure [15], even for simple communicable disease like the Kala-azar, people who are bearing catastrophic medical expenditures [16] due to expensive private care and higher costs for medicines. To address these problems, there are different approaches like community-based health insurance [17], free health services [18, 19], community drug programs and subsidy to disadvantaged and minority populations. However, all of these initiatives have been piloted at different times in the past and have not established a successful model. Therefore, there is need to think of UHC in a different way by designing a scheme for financial protection that covers to all marginalized population, quality health services, and provides comprehensive challenges including the new and re-emerging diseases.

Since 1950, Nepal has been profoundly able to increase the health status and in South East Asia all aspects of health care have been improved but still there are some challenges. From 1950 to 1990, there was a great challenge to extend primary health care, from 1990 to 2006, there were challenges regarding health service integration and after 2006, there have been cumulative challenges of service extension, integration, quality health service, equity, access and financial protection during illness [20]. The People’s Movement, in 2006, established agendas for quality health services, accessible to everyone and guaranteed by the constitution to remove reiterations and improve service delivery [21]. At this date, vital health indicators like life expectancy is 71.5 years, infant mortality rate (IMR) is 29.40/1000 live birth, total fertility rate (TFR) 2.1 children per women and population growth rate 1.74% [22]. In remote areas, the weight of children is less than national average [23]. Now, the constitution of Nepal legally assured health as fundamental right but in practice it may take a long time to achieve it. Nepal has been struggling to expand financial protection in the case of illness for a long period. Since 2016/17, the government of Nepal, Ministry of Health and Population (MoHP) has started social health insurance scheme in some districts. It will be extended in 22 other districts by the end of 2018. Amidst of Nepal’s effort in expanding insurance coverage, this study was done to assess the challenges and opportunities on the road towards Universal Health Coverage in Nepal. Our review will potentially contribute in the national effort to achieve UHC.

## Methodology

### Search strategy to acquire the sources

Search strategies to identify studies regarding UHC in Nepal included searching Google, Google Scholar, PubMed, WHO research portal; the Health Inter-Network Access to Research Initiative (HINARI) and web page of Ministry of Health and Population (MoHP). We applied all of the following key words: ‘universal health coverage’, ‘insurance’, ‘social insurance’, health service, health service coverage, financing, financial protection, legal assurance in health care, ‘Nepal’ in conjugation with boolean operators (AND, OR) in PubMed (opportunity[All Fields] AND (“Plan Parent Chall”[Journal] OR “challenges”[All Fields]) AND universal[All Fields] AND (“health”[MeSH Terms] OR “health”[All Fields]) AND (“AHIP Cover”[Journal] OR “coverage”[All Fields]) AND (“Nepal”[MeSH Terms] OR “Nepal”[All Fields]).

We additionally included equivalent terms from medical subject headings such as ‘Social Security’, ‘Insurance’, ‘Insurance coverage’. As policy documents in general, neither covered in PubMed/Medline nor published electronically elsewhere, we further expanded our search to the web pages of the Ministry of Health and respective departments. For the Scholar Google we used the above terminology and fixed the search by date (almost after 2010) and relevance (proximity to the terms).

Global research related to health for specific country was found in Health Inter-Network Access to Research Initiative (HINARI) and we used this portal too and fixed the search with WHO regional sites (South East Asia), content type (publication and guideline) and all available formats. The selected articles reference list were the potential sources for this study as bibliographical search. The remaining source was taken from Google search as grey materials. The search approaches were targeted on UHC indicators, index and financial protection indicators like out of pocket expenditure, catastrophic health cost, government health spending, total health expending, etc.

**Fig. 1:**
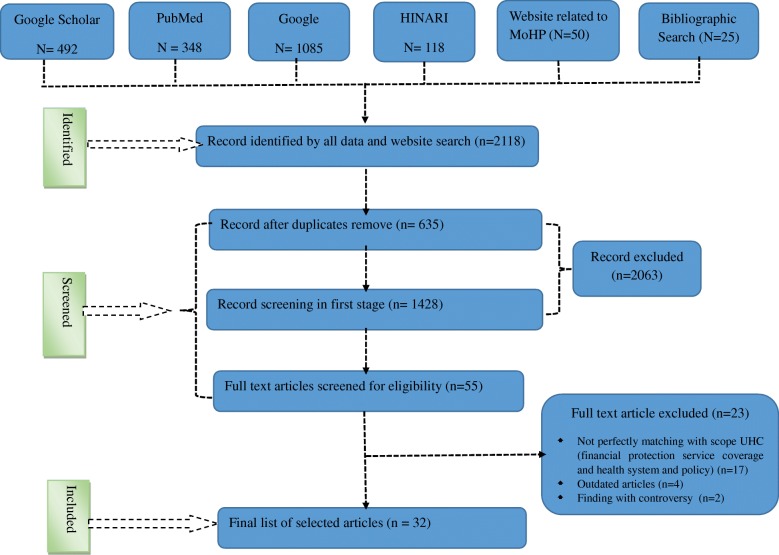
PRISMA flow diagram indicating the study selection procedure on challenges and opportunities towards the road of UHC to include into systematic review, Nepal, 2018

In the first stage, we found 2118 records from Scholar Google, PubMed/Medline, HINARI, web pages of Ministry of Health and Population and its branches and Google search which met the inclusion criteria. In first stage screening we removed 2063 sources due to record duplication and title twisted. We assessed 55 full text articles and excluded 23 sources (due to imperfectly matching the scope with UHC - 17, outdated − 4 and controversial findings − 2). Finally we identified 32 perfectly matched sources for this study (Fig. 1: PRISMA follow chart).

### Inclusion and exclusion criteria

The inclusion criteria for data search were: related to Nepal, with the scope of Universal Health Coverage (wide and operational definitions), usually published since 2010. In wide concept of UHC all types of health services; preventive, promotional, curative, rehabilitative and palliative and in operational term 16 UHC health service indicators and area related to financial protection. The final selection of articles at this stage was based on the following criteria, i) content relevance to the theme of the sources (health service delivery systems, health financing, health insurance, health service quality, etc.), and ii) detail scope for Nepal. Items irrelevant to Nepal, specific health slogans and campaigns, sources from unpublished data were excluded from study.

## Results

We found 32 resources related to the challenges and opportunities for UHC in Nepal where there could be 3 dimensions on challenges and opportunities viz. “legal assurance, risk pulling and financing of health service”; “UHC service coverage status” and “government stewardship, health system and governance on health care in Nepal”. We found 14 research articles related to legal assurance, risk pulling and financing of health service in Nepal. Likewise 11 articles are associated to service coverage status to the scope of UHC and remaining 7 sources are categorized under stewardship, health system and governance on health care in Nepal (Tables 1, 2 and 3).

**Table 1:** List of studies for legal assurance, risk pulling and financing of health service in Nepal 2002–2018: a systematic review, Nepal, 2018

S.N.	Study/sources	Challenges	Opportunities
1	Interim constitution of Nepal [42]	The bill related to health insurance has amended but preparing the modality and scheme became very complex.	Health is accepted as fundamental human right and government must provide basic health service to all citizens.
2	National Health Insurance Policy 2013 [43]	There are many interest groups to dismiss the social health insurance program in Nepal.	Platform has been established to freely discuss the impact of social health insurance.
3	Pokhrel Rajani and Silwal Puskar. Social health insurance in Nepal: A health system departure toward the universal health coverage [44]	Very poor coverage (5% population coverage) of social health insurance in Nepal.	Social health insurance program has been extended and benefit package has been revised.
4	Review of community-based health insurance initiatives in Nepal [45]	Isolated, localized CBHI schemes, as presently implemented in Nepal, do not constitute well accepted model on which national health insurance could be successfully raised.	The positive environment for health insurance has been created by public and health experts.
5	National health insurance policy in Nepal: challenges for implementation [46]	The enrollment status is very low and health workers have no special training.	Training for financial management, application of information technology has been started in hospital health insurance.
6	Implementing a Participatory Model of Micro Health Insurance among Rural Poor with Evidence from Nepal [47].	The participation is volunteer based, premium amount could not pay by poor people and high dropout in current enrollment.	The health insurance is toward mandatory health insurance to all citizen and foreigners too.
7	A Comparative Study on Outcome of Government and Co-Operative Community-Based Health Insurance in Nepal [48]	There is poor awareness on contribution based health service in Nepal.	Community organization like Co-Operative groups can be utilized for universal health insurance coverage.
8	Progress Report on Opportunities, Challenges, Lessons Learned and Strategic Directions for the Implementation of the Nepal Health Sector Programme-2 [49]	There are not enough discussion with interest groups about the health insurance service and payment models.	The social health insurance manual is under discussion with the different stakeholders.
9	E Saito et. al Catastrophic household expenditure on health in Nepal: a cross-sectional survey [50]	The catastrophic health spending is more (13%) than threshold level (10%) in household level and majority of causes are road traffic accident and foreign employment.	Health insurance program by hospital and coverage area has been expanded and disease specific health insurance program has been started.
10	Uprety Sudeep and Lamichhane Bipul 2016. Health Budgeting and Financing in Nepal: Policy Perspectives [51]	There is no assurance for sufficient financing, equitable and efficient resources and financial management and accountability.	Health financing policy discourse has been started.
11	The Current Trade Union Situation in Nepal [52]	Seriously misleading of trade union as a sister organization of political parties and diverting the goal of labor organizations ignoring the issue of financial protection during illness.	Trade Union could strongly advocate the mandatory health insurance of labors and their families to their companies
12	Nepal Labour Market Profile 2014 [53]	Informal market is the main challenges to collect health insurance premium.	Large numbers of youth are outside the countries and can be converted into formal labor market.
13	There is high priority of Universal health coverage in UN sustainable development goal globally [54]	There is no clear paradigm shift to achieve UHC and international support modality has not designed clearly.	There is a global pressure and environment to achieve UHC.
14	Health Federalism: The Role of Health Care Professionals in Nepal [55]	The high amount of out-of-pocket expenditure is increasing due to conflict of interest in health care system and professionals who involve government health system of Nepal.	Health professional councils could involve making health care system like Universal Health Coverage.

**Table 2:** List of the studies for UHC service coverage status in Nepal 2012–2018: a systematic review, Nepal, 2018

SN	Study/sources	Challenges	Opportunities
1	Hogan DR et.al. Monitoring universal health coverage within the Sustainable Development Goals: development and baseline data for an index of essential health services [8]	Nepal’s UHC service coverage index is low i.e. only 46 (The higher rank is > 80) and increasing the UHC service coverage index is challenging.	The legal status is progressive and high probability to accelerate the service coverage.
2	Barriers to using skilled birth attendants’ services inmid- and far-western Nepal: a cross-sectional study [56]	Education level, geographical difficulties and lack of trained human resources are the barrier to achieve SBA coverage.	Political commitment to promote maternal and child health service and conditional cash transfer (CCT) for antenatal checkup institutional delivery and post-partum period from local provincial and central government.
3	Maternal Health in Nepal Progress, Challenges and Opportunities [57]	Lack of awareness about maternal health services, underutilization of maternal health services, social disparities in maternal health, political instability, and low socio-economic status of women, teenage marriage and early pregnancy, unsafe abortion, maldistribution of human resource for health, unavailability and unaffordability of quality care, superstition and indigenous practice.	Special programs have been formulated to achieve the targets of Second Long Term Health Plan, National Health Policy, Millennium Development Goal, and National Health Sector Program and so on.
4	K Sceammell et. al; A landscape analysis of universal health coverage for mothers and children in South Asia [58]	Health facilities have not enough space, privacy, equipment and drugs for maternal and child health service.	Government of Nepal provided high priority to establish well equipped birthing centers in each health facilities.
5	Bhutta ZA et. al Global experience of community health workers for delivery of health related millennium development goals: a systematic review, country case studies, and recommendations for integration into national health systems. [59]	Insufficiency of experts (Physician, Surgeon, Gynecologist etc.) middle and basic level health workforce paramedics and nurse) related to universal health coverage.	Regional and zonal hospitals are going to establish to train health workers.
6	Success factors for women’s and children’s health: Nepal [60]	Inequality of MCH program, poor quality of skill delivery in remote area and increasing poverty after earthquake 2015.	The successful achievement of maternal health (ANC and SBA) and child health in Millennium Development Goal.
7	NK Raut. Path to Universal Health Coverage in Nepal: Is it Achievable? [61]	About 1/3rd of population in rural, mid and far western and poor people have less than adequate basic health service coverage, child vaccination coverage is > 85% and basic maternal health service coverage is about 50% which is due to geographical difficulties, complex bureaucratic structure and not able to define poor and minorities.	Government of Nepal is initiating basic health service package and minimum service standard of each health facilities to achieve UHC with legal and institutional framework.
8	GP Bhandari et.al. State of non-communicable diseases in Nepal [62]	High proportion of NCDs (CVD, COPD, DM and cancer) in non-specialist institutions revels 31% that makes threats to control	It has been high priority program and controlling guidelines and protocols are preparing in community level too.
9	Sharma SR, et.al. Non-communicable disease prevention in Nepal: systemic challenges and future directions [63]	Behavioral factors such as tobacco use, alcohol consumption, physical inactivity and unhealthy diet are driving the epidemic of NCDs, which are further influenced by social, economic and environmental determinants.	Multispectral Action Plan for Prevention and Control of NCDs 2014–2020 has been formulated in grass root level too.
10	Shrestha A. et.al. Water Quality, Sanitation, and Hygiene Conditions in Schools and Households in Dolakha and Ramechhap Districts, Nepal [64]	Quality of water sanitation and hygiene (WASH) is poor in school and household level and maintenance of public toilets have created problems.	School led total sanitation and community led total sanitation program has been started.
11	Verma SC et.al. Prevalence of pulmonary tuberculosis among HIV infected persons in Pokhara, Nepal [65]	Tuberculosis and HIV co-infection, alcohol consumption with TB and low case finding rate in rural area are challenging.	Directly Observed Treatment Short course is still effective and female community health volunteer program accelerates case findings in community.

**Table 3:** List of the studies on government stewardship, health system and governance on health care in Nepal, 2009–2018: a systematic review, Nepal, 2018

*S.N*	Study/sources	Challenges	Opportunities
1	Nepal Millennium Development Goals-Progress Report 2013 [66]	Political instability, poor quality of health service and institutional capacity on remote health institution and inequality in health sector.	Re-structure of health system to implement new constitution.
2	Present Progress of Information Technology in Health Care System of Nepal [67]	Lack and inconsistency of information technology in health care system of Nepal.	There are demonstration projects on consistent and wide health sector information system (HSIS).
3	Addressing the challenges to health sector decentralization in Nepal: an inquiry into the policy and implementation processes [68]	Centralized and weak management of health system, conflict of different policy objectives, improper coordination between section and department under health ministry, weak legal and institutional framework, unstable health financing.	For health care delivery in federal system an expert team has been established and exercising organizational and management structure.
4	Assessing fiscal space for health in Nepal [69]	Poor government effort, poor governance in health service delivery, not able to mobilize the internal resources (tax and donation) and insufficient capacity in district level health structure.	Social health insurance operational manual is going to draft.
5	Decentralization and district health services in Nepal: understanding the views of service users and service providers [70]	The low quality of health care in decentralized institution and existing capacity of health workforce is inadequate.	Decentralize system of health in municipality and rural municipality after the amendment of new constitution 2015.
6	Census of Private Hospitals in Nepal [71]	Almost private hospitals are centralize and difficult to transfer in remote areas.	Enough number of hospitals and competitive medical service is increasing.
7	Susan Heydon. Nepal: Primary Health Care, Universal Health Coverage and Foreign Aid (2015) [72]	Foreign aid in health sector of Nepal has been dispersed, inconsistent has not proper coordination.	Health financing policy is in discussion.

Legal guarantee is the first step to move forward universal health coverage. Legal protection is possible after political commitment, policy endorsement and conceptualization of specific program. Constitutional guarantee of health service to all citizens, amendment of health insurance act, discourse on health financing policy, extension of social health insurance are the major breakthrough and possibilities but poor and volunteer type of health insurance and inadequate awareness level on risk pooling approach during illness are major challenges (Table 1).

Another important aspect of UHC is service coverage situation. WHO and WB jointly prepared UHC index compiling 16 indicators in family planning and reproductive health infectious disease, non-communicable diseases and service capacity and access. Legal advancement for health service delivery, extension of birthing centers, production and enhancement of capacity on human resources, conditional cash transfer (CCT) on ANC and institutional service etc. are the potentialities but the poor achievement of the UHC index, an insufficient awareness level on utilization of health services, inadequate space to provide health services, double burden (infectious and non-communicable) of diseases in health care facilities and community and average quality of water sanitation and hygiene are the main challenges for health service coverage in Nepal (Table 2).

It is necessary to have strong government leadership to achieve UHC. External development partners (EDPs) are just supporting in own their interest but the government role would be influential in a wider area. Restructuring of health service for center, province and local level, upgrading of the health information system through online availability, involvement of private health facilities for quality health service are significant positive factors. Political ignorance about health service due to previous service delivery structure, improper coordination among departments and divisions under MoHP, poor dynamism in health system and donor dependent health financing approach, etc. are the main factors hindering achievement UHC (Table 3).

## Discussion

In this study, the opportunities and factors hindering achievement of UHC in Nepal were explored. Those challenges are multidimensional. Nepal started an insurance scheme recently after a serious lobby of visionary health care professionals, international organizations and interest groups. However, small community based health insurance (CBHI) have been on the scene since 1990s providing small subsidy to people. The coverage of health insurance was small, new enrollment was limited; renewal of health insurance membership dwindled rapidly after some years of implementation. The service coverage of health care was not satisfactory. The quality of health service and financial protection were inadequate. Grass root level health workers were confused about the changing policy of government like user fee, community drug program, free health service, special health care services to minority groups, etc. and none of them ensures comprehensive package of health service with universal access.

UHC is multidimensional because it’s legal, political, health system and socioeconomic agenda arose at the same time and same way all over the world [24]. In most of the Asian countries there are challenges on how to expand health insurance coverage to informal sector, appropriately designing of benefit packages to current health challenges and quality health services [25]. JJ Mogan et al. concluded that high cost and poor access to health care could be the challenge towards UHC [26]. Chu et.al mentioned that Asian low income countries have poor performance on implementing pre-paid financing mechanisms and adopting social health insurance [27]. Inadequate political commitment and decision making power and poor governance are the main challenges towards implementing UHC as experienced in Chile [28]. Those international experiences coincide in Nepalese context too. There has been no priority in regulating drug prices and quality for all citizens but sufficient quality drug supply are major components for UHC. High medicines prices, substandard and counterfeit medicines and the irrational use of medicines are common challenges in developing countries like Nepal [29]. Further, social stratification is a structural challenge. A developed country like France had already achieved UHC some decade ago, also but social inequality in France was the main factor hindering quality health care [30]. High out-of-pocket expenditure, inadequate insurance coverage, increasing medical cost, inefficient use of scare resources, haphazard distribution of resources to the service provider, unequal provision of subsidy to the provinces are main challenges in China [31]. Burden of disease in communicable, infectious and reproductive health, poor availability of trained human resources in health [32], inadequate research to achieve health-care for all [33], commercialized, fragmented, and unregulated health-care delivery systems [34], inequalities in access to health-care, imbalance in resource allocation, high out of pocket health expenditures [35], rising ageing population, social determinants of health such as poverty, illiteracy, alcoholism etc. [36], are main challenges in India. Adequate and only well trained human resources in health can provide quality health services. The gap in human resources is 2 times higher in the African region compared to global average [37]. The experiences of Latin American countries showed that reducing of OPP is a main facilitator for financial protection of people [38]. Those international experiences are in line with Nepal’s context.

Financial protection of health care and economic sustainability are interlinked with each other but there has not been enough discussion on it. The health insurance coverage potentially contributes the sustainable economic growth and economic empowerment contributes for SDG and prosperity [39]. There is a hidden fact that, there was economic boom (two folds economic growth than before UHC) in South Korea, Singapore and Thailand after achieving UHC [40]. WHO has identified that poor government stewardship, governance and health delivery system are the main challenges in developing countries [41] and the situation is also similar in Nepal. Further, production, deployment and monitoring human resource for health could be the milestone to achieve UHC in a stipulated time and social equality is possible after high level political commitment and solidarity of people.

## Conclusion

This is a crucial time to take action for UHC in Nepal because the political system has shifted and the UN SDG is highly focused on UHC in health related goals. Of course, there are some challenges to achieving UHC but those challenges can be addressed with high level political commitment and a businesslike accountable workforce. Population coverage for quality care and financial protection would be major breakthroughs to achieve UHC. Government stewardship, support of stakeholders, policy contribution of experts can only speed up the path towards UHC in Nepal.

## Abbreviations

ANC: Antenatal check-up
CBHI: Community based health insurance
CCT: Conditional cash transfer
EDP: External development partners
HINARI: Health Inter-Network Access to Research Initiative
IMR: Infant mortality rate
IPAID: Institute for Poverty Alleviation and International Development
OECD: Organization for Economic Cooperation and Development
OPP: Out of pocket payment
SBA: Skill birth attendance
SDG: Sustainable development goal
TFR: Total fertility rate
UHC: Universal health coverage
UN: United Nations
WB: World Bank
WHO: World Health Organization

## References

[1] World Health Organization (2016). Health financing for universal health coverage.

[2] Organization WH. Tracking universal health coverage: first global monitoring report: World Health Organization, Geneva; 2015.

[3] Clark J. Medicalization of global health 4: the universal health coverage campaign and the medicalization of global health. Glob Health Action. 2014;7. 10.3402/gha.v7.24004. 10.3402/gha.v7.24004 PMC4028903 24848662

[4] Stuckler D, Feigl AB, Basu S, McKee M (2010). The political economy of universal health coverage. Background paper for the global symposium on health systems research.

[5] Bärnighausen T, Sauerborn R (2002). One hundred and eighteen years of the German health insurance system: are there any lessons for middle-and low-income countries?. Soc Sci Med.

[6] World Health Organization: Tracking universal health coverage: 2017 global monitoring report. 2017.

[7] (ADDCN) AoDDCoN (2008). State restructuring and issues of local self governance in Nepal.

[8] Hogan DR, Stevens GA, Hosseinpoor A, Ranabhat CL (2018). Monitoring universal health coverage within the sustainable development goals: development and baseline data for an index of essential health services. Lancet Glob Health.

[9] Sustainable Development Goals [http://www.un.org/sustainabledevelopment/sustainable-development-goals/]. Accessed Mar 2018.

[10] Ranabhat CL, Atkinson J, Park M-B, Kim C-B, Jakovljevic M (2018). The influence of universal health coverage on life expectancy at birth (LEAB) and healthy life expectancy (HALE): a multi-country cross-sectional study. Front Pharmacol.

[11] Ranabhat CL, Park M-B, Kim C-B, Kim C-S, Jeong H-S, Koh SB, Chang S-J. Influence of key health related indicators on adult mortality: result from UN member countries. Iran J Public Health. 2018;47(6):794. PMC6077630 30087864

[12] Ranabhat CL, Kim C-B, Park M-B, Acharaya S (2017). Multiple disparities in adult mortality in relation to social and health care perspective: results from different data sources. Glob Health.

[13] Xu K, Evans DB, Carrin G, Aguilar-Rivera AM, Musgrove P, Evans T (2007). Protecting households from catastrophic health spending. Health Aff.

[14] Department of Health Service Kathmandu Nepal (2015). Annual report, DoHS Kathmandu.

[15] Data [http://data.worldbank.org/topic/health]. Accessed Mar 2018.

[16] Adhikari SR, Maskay NM, Sharma BP (2009). Paying for hospital-based care of kala-azar in Nepal: assessing catastrophic, impoverishment and economic consequences. Health Policy Plan.

[17] Community Based Health Insurance Practices in Nepal [https://www.academia.edu/5587577/Community_Based_Health_Insurance_Practices_in_Nepal]. Accessed Apr 2018.

[18] Mahato PK, Sharma Paudel G (2015). Access to free health-care services for the poor in tertiary hospitals of western Nepal: a descriptive study. WHO South East Asia J Public Health.

[19] Adhikari SR (2013). An evaluation of Nepal’s free health care schemes: evidence from a quasi-experimental design.

[20] Dixit H. Nepal’s quest for health: the health services of Nepal: Educational Publishing House, Kathmandu; 2014.

[21] Government of Nepal, Population MoHa. The sector-wide approach in the health sector: achievement and lesson learn. Research Triangle Park; 2010.

[22] Health in Nepal [https://en.wikipedia.org/wiki/Health_in_Nepal]. Accessed Apr 2018.

[23] Ranabhat C, Kim C-B, Park M, Kim C, Freidoony L (2016). Determinants of body mass index and intelligence quotient of elementary school children in mountain area of Nepal: an explorative study. Children.

[24] McKee M, Balabanova D, Basu S, Ricciardi W, Stuckler D. Universal Health Coverage: A Quest for All Countries but under Threat in Some. Value Health. 2013;16 (1, Supplement):S39–45. 10.1016/j.jval.2012.10.001 23317643

[25] Bredenkamp C, Evans T, Lagrada L, Langenbrunner J, Nachuk S, Palu T (2015). Emerging challenges in implementing universal health coverage in Asia. Soc Sci Med.

[26] Mongan JJ (2007). Health financing: challenges and opportunities, coverage and cost. Wanting it all: the challenge of reforming the US health care system.

[27] Chu A, Kwon S, Cowley P. Health financing reforms for moving towards universal health coverage in the western pacific region. Health Syst Reform. 2018; (just-accepted). 10.1080/23288604.2018.1544029 30924747

[28] Saavedra M, Greer S, Méndez C (2015). Governance, decision-making, and universal health coverage: perceptions from Chilean health decision-makers. Value Health.

[29] Bigdeli M, Laing R, Tomson G, Babar Z-U-D (2015). Medicines and universal health coverage: challenges and opportunities. J Pharm Policy Prac.

[30] Nay O, Béjean S, Benamouzig D, Bergeron H, Castel P, Ventelou B. Achieving universal health coverage in France: policy reforms and the challenge of inequalities. Lancet. 2016;387(10034):2236–49. 10.1016/S0140-6736(16)00580-8 27145707

[31] Hu S, Tang S, Liu Y, Zhao Y, Escobar M-L, de Ferranti D. Reform of how health care is paid for in China: challenges and opportunities. Lancet. 2008;372(9652):1846–53. 10.1016/S0140-6736(08)61368-9 18930520

[32] Rao M, Rao KD, Kumar AK, Chatterjee M, Sundararaman T (2011). Human resources for health in India. Lancet.

[33] Dandona L, Raban MZ, Guggilla RK, Bhatnagar A, Dandona R (2009). Trends of public health research output from India during 2001-2008. BMC Med.

[34] Agarwal D (2012). Universal access to health care for all: exploring road map. Indian J Community Med.

[35] Balarajan Y, Selvaraj S, Subramanian SV (2011). Health care and equity in India. Lancet (London, England).

[36] Health CoSDo (2008). Closing the gap in a generation: health equity through action on the social determinants of health: final report of the commission on social determinants of health.

[37] Sambo LG, Kirigia JM (2014). Investing in health systems for universal health coverage in Africa. BMC Int Health Hum Rights.

[38] Titelman D, Cetrángolo O, Acosta OL. Universal health coverage in Latin American countries: how to improve solidarity-based schemes. Lancet. 2015;385(9975):1359–63. 10.1016/S0140-6736(14)61780-3 25458734

[39] Russo G, Bloom G, McCoy D (2017). Universal health coverage, economic slowdown and system resilience: Africa’s policy dilemma. BMJ Global Health.

[40] GDP growth annual [https://data.worldbank.org/indicator/NY.GDP.MKTP.KD.ZG]. Accessed May 2018.

[41] World Health Organization. Health systems governance for universal health coverage action plan. Geneva: Department of Health Systems Governance and Financing; 2014. p. 14040–902.

[42] Secretariat CA, Durbar S, Nepal Po (2015). Constitution of Nepal 2015.

[43] Ministry of Health and Population, Service DoH (2013). National Health Insurance Policy-2013.

[44] Pokharel R, Silwal PR. Social health insurance in Nepal: a health system departure toward the universal health coverage. Int J Health Plann Manag. 2018. 10.1002/hpm.2530. 10.1002/hpm.2530 29635799

[45] Stoermer M, Fuerst F, Rijal K, Bhandari R, Nogier C, Gautam GS, Hennig J, Hada J, Sharma S (2012). Review of community-based health insurance initiatives in Nepal. Deutsche Gesellschaft fur internationale Zusammenarbeit (GIZ) Gmbh.

[46] Mishra SR, Khanal P, Karki DK, Kallestrup P, Enemark U. National health insurance policy in Nepal: challenges for implementation. Glob Health Action. 2015;8. 10.3402/gha.v8.28763. 10.3402/gha.v8.28763 PMC4546934 26300556

[47] Dror MD, Majumdar A, Panda P, John D, Koren R (2014). Implementing a participatory model of micro health insurance among rural poor with evidence from Nepal. Geneva Pap Risk Insur Issues Pract.

[48] Ranabhat CL, Kim C-B, Singh DR, Park MB (2017). A comparative study on outcome of government and co-operative community-based health insurance in Nepal. Front Public Health.

[49] Path R, Kathmandu N (2014). Progress report on opportunities, challenges, lessons learned and strategic directions for the implementation of the Nepal health sector Programme-2.

[50] Saito E, Gilmour S, Rahman MM, Gautam GS, Shrestha PK, Shibuya K (2014). Catastrophic household expenditure on health in Nepal: a cross-sectional survey. Bull World Health Organ.

[51] Lamichhane SUB (2016). Health budgeting and financing in Nepal: policy perspectives.

[52] Dahal DR. The current trade union situation in Nepal: Friedrich Ebert Stiftung Foundation, Kathmandu; 2002.

[53] Danish Trade Union Council of International Development Cooperation (2014). Nepal – Labour Market Profile 2014.

[54] Buse K, Hawkes S (2015). Health in the sustainable development goals: ready for a paradigm shift?. Glob Health.

[55] Dulal RK (2009). Health federalism: the role of health care professionals in Nepal. J Nepal Med Assoc.

[56] Choulagai B, Onta S, Subedi N, Mehata S, Bhandari GP, Poudyal A, Shrestha B, Mathai M, Petzold M, Krettek A (2013). Barriers to using skilled birth attendants’ services in mid-and far-western Nepal: a cross-sectional study. BMC Int Health Hum Rights.

[57] Bhusal C, Bhattarai S, Bhaskar RK (2015). Maternal health in Nepal progress, challenges and opportunities. Int J Med Health Res.

[58] Scammell K, Noble DJ, Rasanathan K, O'Connell T, Ahmed AS, Begkoyian G, Goldner T, Jayatissa R, Kuppens L, Raaijmakers H (2016). A landscape analysis of universal health coverage for mothers and children in South Asia. BMJ Global Health.

[59] Bhutta ZA, Lassi ZS, Pariyo G, Huicho L (2010). Global experience of community health workers for delivery of health related millennium development goals: a systematic review, country case studies, and recommendations for integration into national health systems. Glob Health Workforce Alliance.

[60] World Health Organization. Success factors for women’s and children’s health. Nepal. Publications of the World Health Organization, Geneva; 2015.

[61] Raut NK. Path to universal health coverage in Nepal: is it achievable? National Graduate Institute for Policy Studies, Kathmandu; 2015. p. 1–17.

[62] Bhandari GP, Angdembe MR, Dhimal M, Neupane S, Bhusal C (2014). State of non-communicable diseases in Nepal. BMC Public Health.

[63] Sharma SR, Page R, Matheson A, Lambrick D, Faulkner J, Mishra SR. Non-communicable disease prevention in Nepal: systemic challenges and future directions. Glob Health Promot. 2017:1757975917720800. 10.1177/1757975917720800. 10.1177/1757975917720800 28862520

[64] Shrestha A, Sharma S, Gerold J, Erismann S, Sagar S, Koju R, Schindler C, Odermatt P, Utzinger J, Cissé G (2017). Water quality, sanitation, and hygiene conditions in schools and households in Dolakha and Ramechhap districts, Nepal: results from a cross-sectional survey. Int J Environ Res Public Health.

[65] Verma SC, Dhungana GP, Joshi HS, Kunwar HB, Pokhrel AK (2012). Prevalence of pulmonary tuberculosis among HIV infected persons in Pokhara, Nepal. J Nepal Health Res Counc.

[66] Pokharel JC, Pradhan HK, Hada B, Kumar BR, Chaudhary RP (2013). Nepal millennium development goals-progress report 2013.

[67] SHRESTHA MR (2014). Present progress of information technology in health care system of Nepal. Jpn Med Assoc J.

[68] Dhakal R, Ratanawijitrasin S, Srithamrongsawat S (2009). Addressing the challenges to health sector decentralization in Nepal: an inquiry into the policy and implementation processes. Nepal Med Coll J.

[69] Belay T, Tandon A (2011). Assessing fiscal space for health in Nepal.

[70] Regmi K, Naidoo J, Pilkington PA, Greer A (2010). Decentralization and district health services in Nepal: understanding the views of service users and service providers. J Public Health.

[71] Center Beureo of Statistics. Census of private hospitals in Nepal. Central Bureau of Statistics Thapathali, Kathmandu; 2013.

[72] Heydon DS (2015). Primary Health Care, Universal Health Coverage and Foreign Aid: Nepal. The University of York 2015: Centre for Global Health Histories.

